# Enhanced skin cancer diagnosis: a deep feature extraction-based framework for the multi-classification of skin cancer utilizing dermoscopy images

**DOI:** 10.3389/fmed.2024.1495576

**Published:** 2024-11-13

**Authors:** Hadeel Alharbi, Gabriel Avelino Sampedro, Roben A. Juanatas, Se-jung Lim

**Affiliations:** ^1^College of Computer Science and Engineering, University of Hail, Ha'il, Saudi Arabia; ^2^Department of Computer Science, University of the Philippines Diliman, Quezon, Philippines; ^3^College of Computing and Information Technologies, National University, Manila, Philippines; ^4^School of Electrical and Computer Engineering, Yeosu Campus, Chonnam National University, Gwangju, Republic of Korea

**Keywords:** skin cancer diagnosis, feature extraction, multi-classification, dermoscopy images, deep learning, meta-data, lesion

## Abstract

Skin cancer is one of the most common, deadly, and widespread cancers worldwide. Early detection of skin cancer can lead to reduced death rates. A dermatologist or primary care physician can use a dermatoscope to inspect a patient to diagnose skin disorders visually. Early detection of skin cancer is essential, and in order to confirm the diagnosis and determine the most appropriate course of therapy, patients should undergo a biopsy and a histological evaluation. Significant advancements have been made recently as the accuracy of skin cancer categorization by automated deep learning systems matches that of dermatologists. Though progress has been made, there is still a lack of a widely accepted, clinically reliable method for diagnosing skin cancer. This article presented four variants of the Convolutional Neural Network (CNN) model (i.e., original CNN, no batch normalization CNN, few filters CNN, and strided CNN) for the classification and prediction of skin cancer in lesion images with the aim of helping physicians in their diagnosis. Further, it presents the hybrid models CNN-Support Vector Machine (CNNSVM), CNN-Random Forest (CNNRF), and CNN-Logistic Regression (CNNLR), using a grid search for the best parameters. Exploratory Data Analysis (EDA) and random oversampling are performed to normalize and balance the data. The CNN models (original CNN, strided, and CNNSVM) obtained an accuracy rate of 98%. In contrast, CNNRF and CNNLR obtained an accuracy rate of 99% for skin cancer prediction on a HAM10000 dataset of 10,015 dermoscopic images. The encouraging outcomes demonstrate the effectiveness of the proposed method and show that improving the performance of skin cancer diagnosis requires including the patient's metadata with the lesion image.

## 1 Introduction

Skin cancer has become one of the most prevalent and widely dispersed types of cancer worldwide in recent decades. It is a prevalent form of cancer that starts with an overabundance of skin cells proliferating. It can be caused by UV radiation from sunlamps and tanning beds, which encourages the proliferation of skin cells and the formation of cancerous tumors (1–3). One of the major causes of death worldwide is skin cancer. It was predicted that by 2023, 97,160 Americans would receive a skin cancer diagnosis, accounting for 5.0% of all cancer cases registered in the US, and 7,990 Americans passed away from skin cancer, accounting for 1.3% of all skin cancer-related deaths in the US (4). A common skin cancer that can spread fast to other parts of the body and even be deadly is called melanoma. Between 2016 and 2020, the US had about 21 cases of melanoma out of 100,000 cases reported. Melanoma claimed the lives of 1,413,976 persons in 2020, with a mortality rate of 2.1 per 100,000 diagnosed cases. Skin melanoma has a comparatively high 5-year survival rate of 93.5%. Early detection of cutaneous melanoma results in a 99.6% 5-year survival rate (4). Only 77.6% of skin melanomas are discovered at the local stage, despite the fact that localized skin melanoma has a higher probability of survival since it does not migrate to other body areas. If skin melanoma is discovered early on, the frequency of deaths from it can be decreased (5).

The development of computer-aided detection systems for skin cancer diagnosis was necessitated by the limits of dermoscopy and the need for improved accuracy in skin cancer diagnosis (3, 6). Expert dermatologists have an accuracy rate of over 60% when diagnosing melanoma without the use of specialized visual aids (7). In both medical and non-medical domains, however, performance has improved due to the latest advancements in deep learning-based techniques. They can also help dermatologists adhere to skin lesions in images to identify cancer early. The three common techniques used to apply imaging for skin lesions are histological, photographic, and dermoscopic images. High-resolution skin imaging is achieved through the use of specialized equipment to obtain dermoscopy images with a decrease in skin surface reflectance (8). Histological images are obtained using invasive biopsies and microscopy (7). Simple photographic images from smartphones and cameras can have inconsistent illumination, zoom, and perspective, as well as irrelevant backgrounds. In contrast, high-quality standardized images are produced via dermoscopy and histology methods (9). This significantly increases the difficulty of automatic classification. By using millions of pre-training and training images, a data-driven approach to solving this issue makes classification resistant to photographic variability (10).

### 1.1 Motivation

Dermatologists diagnose skin cancer by differentiating benign and malignant lesions that have the same source and similar shape, border, and color despite the fact that both are composed of melanocyte cells. This is a challenging endeavor. Differentiating between malignant keratinocyte carcinoma (BCC) and benign keratosis (SCC) is very challenging (8). Malignant cutaneous lymphomas and inflammatory non-neoplastic dermatitis can be difficult to differentiate from one another. Furthermore, it can be difficult to distinguish between benign and malignant dermal lesions, such as Kappi sarcoma and dermatofibroma and vascular lesions (8). Given the high error rate associated with ocular inspection, biopsies, and histological investigations represent the gold standards of diagnosis. Because of these challenges with diagnosis, dermatologists frequently use biopsies and histological tests as the gold standards to determine which skin disorders are benign and which are malignant. These techniques considerably lower errors in diagnosis and enhance patient outcomes by offering conclusive knowledge that visual inspection alone cannot deliver. The objective of this study is to offer a novel deep-learning method for automatically predicting the type of skin lesion so that doctors may use it as a tool to evaluate skin lesion images. The proposed model also considered patient characteristics, the lesion's anatomical location, age, and gender in order to forecast the kind of lesion. Based on a recent study, males are 4% more likely than women to die from melanoma skin cancer and are 10% more likely to get the condition. The incidence rate of skin cancer also increases with age. There is also evidence of a relationship between the type of lesion and its anatomical location on the body (4, 11). These results prompted us to investigate the effects of providing the automated model for skin cancer diagnosis with this data (age, gender, and anatomical site).

### 1.2 Research contribution

This research renders it feasible to identify human actions more accurately and efficiently. The following lists the main findings and contributions of the research.

- The study proposed four variants of the CNN model: the original CNN, CNN without batch normalization, CNN with few filters, and CNN with strided to categorize and predict skin cancer. The goal of these developments was to optimize the network architecture for higher diagnostic accuracy. The study also proposed novel hybrid models by combining CNN with traditional machine learning classifiers, such as SVM, RF, and LR. This approach combined the benefits of traditional classifiers with DL to boost overall classification accuracy.
- The study provides a thorough EDA with random oversampling approaches to balance and standardize the dataset. The accuracy and generalizability of the model across a variety of skin lesion images were much enhanced by this preprocessing phase. The research made a substantial contribution by integrating patient metadata into the prediction model in addition to lesion images. The outcomes showed that this combination improved classification accuracy by at least 5%, underscoring the significance of considering extra patient data when diagnosing skin cancer.
- The CNN-RF and CNN-LR models obtained 99% accuracy, while the CNN-SVM, original CNN, and strided CNN models got 98% accuracy. The proposed CNN-based methods were able to achieve these astounding accuracy rates. These results demonstrate the effectiveness of the proposed methods in raising the accuracy of skin cancer diagnosis.

### 1.3 Organization

The structure of the article is as follows. Section 2 provides background material and relevant articles. Section 3 provides the proposed method. The effectiveness of the proposed approach is evaluated and compared to the baseline methods in Section 4. Recommendations are given in Section 5 following the article's conclusion.

## 2 Related work

Artificial Intelligence (AI) has great promise for the management of hematologic malignancies in the future because of its ability to interpret data from several diagnostic modalities, estimate mortality, and suggest therapeutic methods. Machine learning-based methods address various medical data and illnesses. According to Eckardt et al. (12), these ML techniques can be applied to a wide range of applications to ensure prompt and precise diagnosis, risk assessment, and efficient treatment.

The authors in Harish et al. (13) offered a comprehensive approach to early skin cancer detection. As part of the preprocessing step, a multi-filter Fusion and Equalization (MFE) is recommended to reduce noise and highlight key features in Digital Dermoscopic (DD) images. For segmentation, a boundary contrast-based Otsu threshold technique (BCOT) is provided to guarantee precise delineation of lesion boundaries. It is imperative to incorporate both spatial linkages and local differences in texture to improve the durability of feature extraction. Providing a more potent and selective feature set that is better able to capture textural and structural information for recognition tasks. This is accomplished by combining the use of the Local Optimal Oriented Pattern (LOOP) approach with the gray-level co-occurrence matrix (GLCM). GLCM, capture intensity-based data and pixel pattern analysis are further improved by LOOP, which enriches the extracted characteristics. The skin lesions are classified in the classification step using the Random Forest (RF) machine learning technique. The research uses a dataset of 800 images from the Kaggle database, which originates from the ISIC and shows both malignant and benign types of oncological disorders. A 98.75% accuracy level was obtained with this strategy. The authors in Mohammed et al. (14) suggested the use of a hybrid model with a support vector machine (SVM) acting as the classifier and DenseNet201 and auto-encoder for feature extraction. Nine distinct classes make up the ISIC 2016 dataset, which was used to evaluate the proposed model. The hybrid model successfully identified nine distinct types of skin cancer with a classification accuracy of 91.09%.

The authors in Packiamary and Muthukumaravel (15) presented a hybrid system for diagnosing melanoma that integrates CNNs and advanced feature extraction with conventional image processing. Convolutional layers are used to learn features, and completely linked layers are used for classification in the CNN architecture, which is specifically designed to classify melanoma. With 99.1% accuracy, 98.3% precision, 96% recall, and 96.5% F1 Score in computational metrics, the results demonstrate that the proposed system performs better than the current systems. The author in study (16) addresses skin cancer by introducing a deep learning-driven automated system, the DenseNet 169 model, which was trained on the extensive Skin Cancer: Malignant vs. Benign dataset. The technology performs exceptionally well in the crucial area of early detection. DenseNet169 has an astounding 89.7% success rate in accurately classifying skin lesions by utilizing dermoscopy images. Authors in Andleeb et al. (17) introduced an attention-based technique based on the premise that the lesion is the most informative region of dermoscopic images; thus, the proposed approach involves lesion localization before categorization. Then, a feature extractor driven by Deep Learning is used to extract the black-box features of the lesion segment, and another feature extractor is used to extract features from the entire image. After concatenating the two feature vectors, a composite feature vector is produced that, when used by a simple neural network, enables image classification. Empirical validation with the proposed methodology on the ISIC 2019 dataset yielded a 75.5% accuracy rate.

Authors in Naeem et al. (6) A unique deep learning-based framework is proposed for the multiclassification of skin cancer types, including basal cell carcinoma, melanoma, melanocytic nevi, and benign keratosis. The proposed model, called SCDNet, classifies various forms of skin cancer by fusing convolutional neural networks (CNN) and Vgg16. The ISIC 2019 dataset is used to assess the performance of the proposed SCDNet classifier. In comparison to Resnet 50, Alexnet, Vgg19, and Inception-v3, which have accuracy rates of 95.21%, 93.14%, 94.25%, and 92.54%, respectively, the proposed SDCNet has an accuracy rate of 96.91% for the multiclassification of skin cancer. Authors in Daghrir et al. (18) proposed SNCNet, which combines features extracted from dermoscopic images using both handmade (HC) and deep learning (DL) models to categorize the eight different forms of skin cancer. A convolutional neural network (CNN) is utilized for classification. Dermoscopy images from the publicly accessible ISIC 2019 dataset are used to train and evaluate the model for the diagnosis of skin cancer. The proposed model outperformed the four baseline models and the SOTA classifiers, with a precision of 98.31%, recall of 97.89%, accuracy of 97.81%, and F1 score of 98.10%.

Authors in Chaturvedi et al. (19) investigated a highly effective automated approach for classifying skin cancer. They used a MobileNet model that was pretrained using about 12,80,000 images from the 2014 ImageNet issues. Transfer learning was utilized in the study to refine the model with 10,015 dermoscopy images from the HAM10000 dataset. The model used in this investigation produced an overall accuracy of 83.1% for seven classes in the dataset; the top two and top three classes achieved accuracy of 91.36% and 95.34%, respectively. Additionally, it was discovered that the weighted averages for recall, precision, and f1-score were, respectively, 89%, 83%, and 83%. The authors in Shapna Akter et al. (20) have presented a number of DL models for the classification of skin lesions to differentiate skin cancer from other forms of skin lesions. Preprocessing and augmentation techniques are applied to the data before the skin lesions are classified. In the end, a CNN model and six transfer learning models are trained on the benchmark HAM10000 dataset to classify seven kinds of skin lesions. For inceptionv3, Xception, Densenet, Mobilenet, Resnet, CNN, and VGG16, the study's accuracy results are 90%, 88%, 88%, 87%, 82%, and 77%, respectively.

In Elshahawy et al. (21), the author proposed a new model for melanoma diagnosis with its degree that combines “you only look once” (YOLOv5) and “ResNet50” and train images (HAM10000). Secondly, feature maps include gradient modifications, which make inference faster, improve accuracy, and reduce the number of hyperparameters in the model, making it smaller. To achieve the intended results, additional classes for dermatoscopic images of common lesions with pigmented skin are added to the existing YOLOv5 model. Average performance measures are 99.0%, 98.6%, 98.8%, 99.5%, 98.3%, and 98.7% for accuracy, recall, mean average precision (MAP) from 0.5 to 0.95, and dice similarity coefficient (DSC) from 0.0 to 0.5, respectively. Authors in Imran et al. (22), a pre-trained CNN combined with a feature optimization technique driven by nature is presented as a skin cancer classification model. The ISIC collection of dermoscopic images is used to create a custom dataset that includes microscopic depictions of both benign and malignant skin cancer. Deep feature extraction and pattern identification are carried out on both upgraded and original dataset images using the pre-trained CNN model EfficientNetB0. The improved feature vector with several SVM classifier kernels is then used to complete the skin cancer classification task. The proposed model retained its high prediction speed and low training duration, and its accuracy reached 98% using CB-SVM.

The applicability of skin cancer prediction research to other datasets' features and classes is restricted by its reliance on specific datasets. The accuracy of the model across a variety of demographic factors is frequently impacted by the lack of diversity among participants. Conventional deep learning models are computationally intensive, data-driven, complicated, and prone to overfitting. Alternatively, potential efficiency, interpretability, dimension reduction, and a strong theoretical base are provided by the variation CNN model. This study uses the four-variant CNN models for skin cancer prediction and classification. Table 1 provides the summary of the literature review.

**Table 1:** Literature review summary.

**References**	**Focus**	**Techniques**	**Results**	**Limitations**
Andleeb et al. (17)	Melanoma detection	Deep learning with attention maps	75.5% accuracy	Limited to melanoma; generalizability to other skin cancers not discussed
Harish et al. (13)	Skin cancer detection	Transfer learning-based hybrid model	92.5% accuracy	Model complexity may affect real-time application and generalization to diverse datasets
Naeem et al. (6)	Skin cancer detection	Integration of handcrafted and deep learning features	93.8% accuracy	Integration may increase computational requirements; limited evaluation on larger datasets
Chaturvedi et al. (19)	Multi-class skin cancer classification	MobileNet-based model	90.7% accuracy	Lower accuracy in rare classes; limited to seven skin lesion types
Shapna Akter et al. (20)	Multi-class skin cancer classification	Deep CNN	91.3% accuracy	Possible overfitting due to deep architecture; lack of evaluation on external datasets

## 3 Material and methods

**Figure 1:**
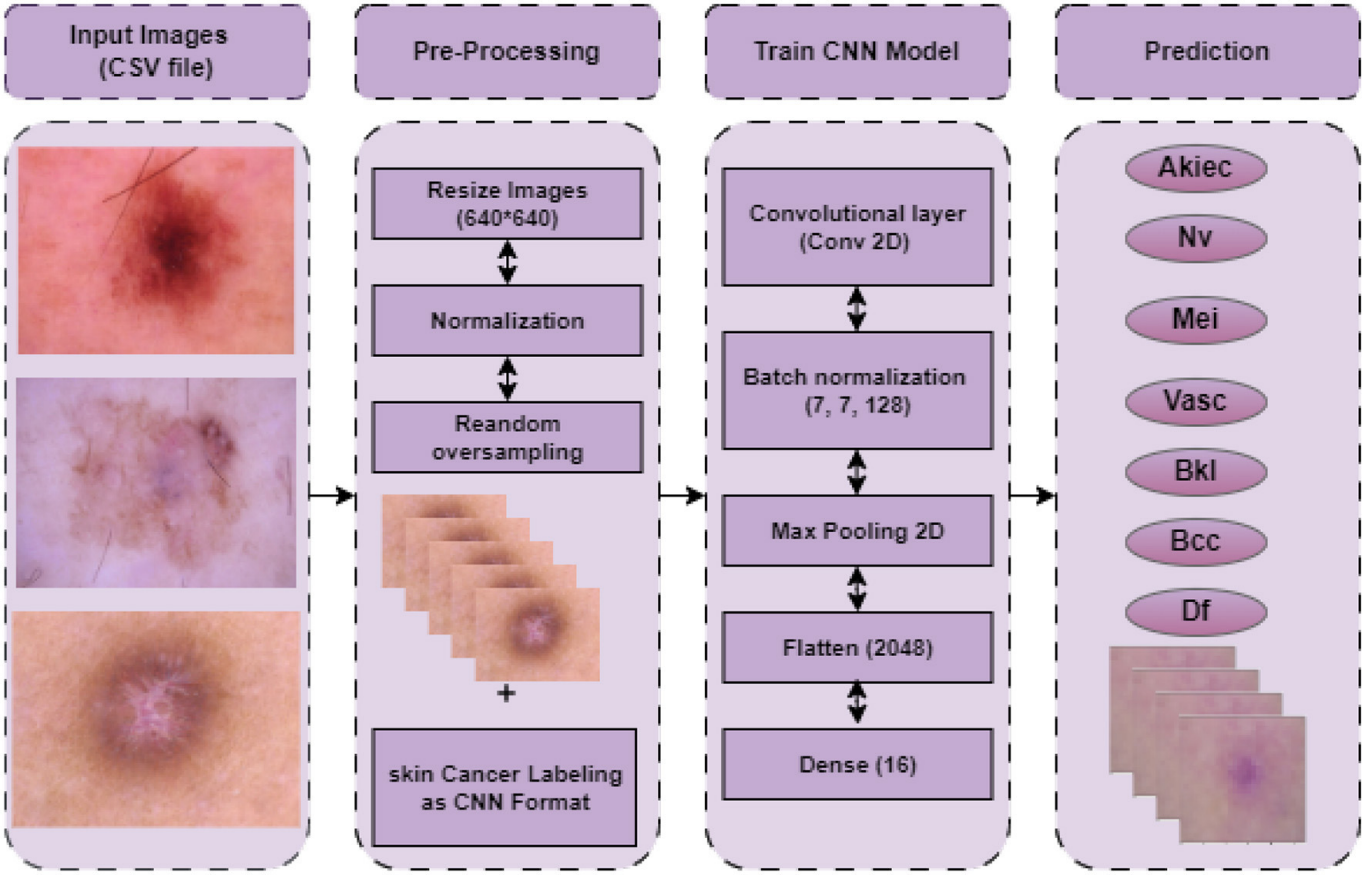
Proposed framework for skin cancer prediction.

This section outlines the entire process of the proposed approach. The proposed approach entails a number of phases, including acquiring datasets, preparing data, and creating model predictions. The procedure for detecting skin cancer with a Convolutional Neural Network (CNN), including all stages involved, from preparing data to making predictions, is presented in Figure 1. The initial step in the skin cancer prediction process is loading disease images. Scaling the images to a standard size of 640 × 640 pixels is a crucial part of pre-processing to maintain consistency across the input data. Subsequently, the pixel values undergo normalization within a designated range (e.g., 0–1) in order to mitigate numerical instability and optimize the training process. Furthermore, by replicating underrepresented images in the dataset, random oversampling is employed to rectify class imbalance and ensure an equitable distribution of training data. After pre-processing, the labeled dataset is used to train the CNN model. The convolutional layer uses filters to extract features from input images; the batch normalization layer stabilizes activations to enhance training and lessen overfitting; and the max-pooling layer, which downsamples feature maps to save computational overhead and dimensionality while maintaining essential features, is the main layer that makes up the CNN architecture. The fully connected layers are fed the feature maps by the flattened layer, which then transforms them into a one-dimensional vector. A dense layer with sixteen neurons then combines the extracted features to generate the classification output. In the last stage, the prediction phase, the model generates a classification result and predicts the type of skin cancer based on the input images.

### 3.1 Data description

**Figure 2:**
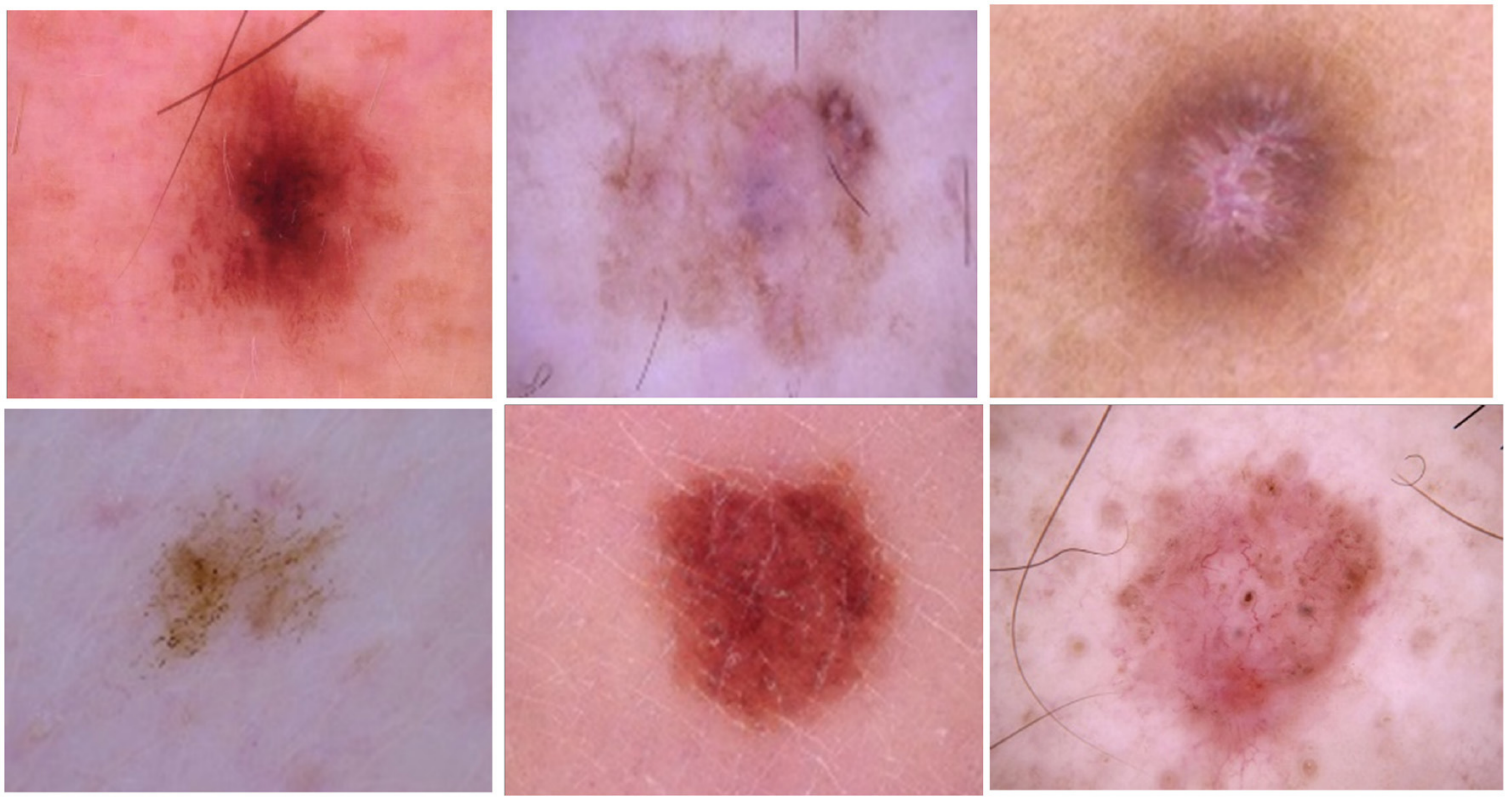
Samples of skin cancer classes.

The HAM10000 (“Human Against Machine with 10,000 training images”) dataset is a sizable compilation of dermatoscopic images of pigmented lesions extracted from multiple sources. Diverse populations provide the dermatoscopic images, which are obtained and archived using various modalities. One potential use for the final dataset is as a training set for academic ML applications, given it includes 10,015 dermatoscopic images. The HAM10000 dataset's images are saved in JPEG format, which offers sharp color information necessary for accurate dermatoscopic assessment. The collection also contains metadata for each entry, which provides useful context for the images. This metadata includes the patient's age and gender, the anatomical site of the lesion (e.g., scalp, face, or trunk), and the kind of diagnosis (e.g., follow-up, expert consensus, or histology). The addition of this metadata improves the relevance and accuracy of skin lesion categorization models. The cases represent all significant diagnostic categories related to pigmented lesions: dermatofibroma (df), melanoma (mel), melanocytic nevi (nv), actinic keratoses and intraepithelial carcinoma/Bowen's disease (akiec), basal cell carcinoma (bcc), benign keratosis-like lesions (solar lentigines/seborrheic keratoses and lichen-planus like keratoses, bkl), melanoma (mel), melanocytic nevi (nv), and vascular lesions (angiomas, angiokeratomas, pyogenic granulomas and hemorrhage, vasc). For the other cases, the basis of truth is established by *in-vivo* confocal microscopy (confocal), expert consensus (consensus), or follow-up inspection (follow_up); lesion discoveries in more than half of the cases are accounted for by histopathology (histo). The samples of skin cancer are represented in Figure 2.

### 3.2 Data preprocessing

Preparing unprocessed data into a sanitized and appropriate format for analysis and modeling is known as data preprocessing. There are several steps involved in improving the quality of the data and preparing it for statistical analysis or machine learning algorithms. Preprocessing is a critical stage since the structure and quality of the incoming data directly impact the functionality of any successor models.

#### 3.2.1 Data cleaning

Data cleaning, an essential phase in data analysis, is getting the raw data ready for analysis by fixing or eliminating erroneous records, making sure the data is consistent, and adding the missing information. The primary objectives are to enhance the quality of the data and prepare it for modeling or additional analysis. There are several steps involved in the data cleaning process. Firstly, to identify the different categories that are present, examine the unique values in the target variable column and separate the target variable from the dataset. A feature matrix is produced by dropping the target variable and identifying the missing values. To make sure there are no null values present, *sum*().*sum*() is utilized. *Metadata*.*head*() gives a peek at the metadata structure. The metadata preview is validated by calling *meta*_*data*.*head*() once again, and the missing values check is performed once more to confirm that there are no null values in the data DataFrame.

#### 3.2.2 Exploratory data analysis

**Figure 3:**
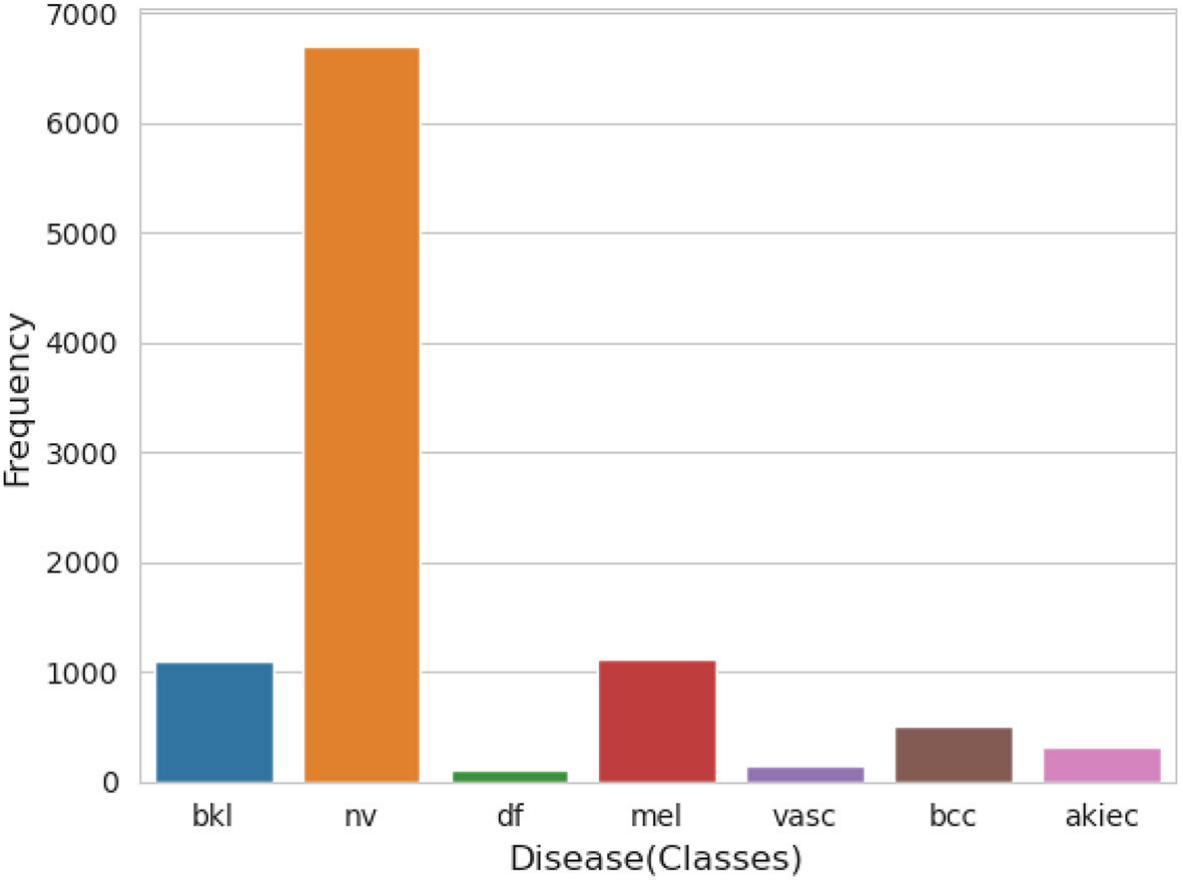
Frequency distribution of classes.

An essential component of any research endeavor is Exploratory Data Analysis (EDA). The primary objective of the exploratory analysis is to identify anomalies and outliers in the data so that it concentrates on testing the hypothesis. It also provides resources for data visualization and analysis, usually through graphical representation, to help in hypothesis generation. After data collection, EDA is completed. The information is effectively plotted, updated, and presented without the need for assumptions in order to assess the quality of the data and build models (23). We applied the EDA technique to the HAM10000 dataset, which has 7 skin cancer classes (i.e., bkl, nv, df, mel, vasc, bcc, and akiec). The frequency distribution of all classes is illustrated in Figure 3.

**Figure 4:**
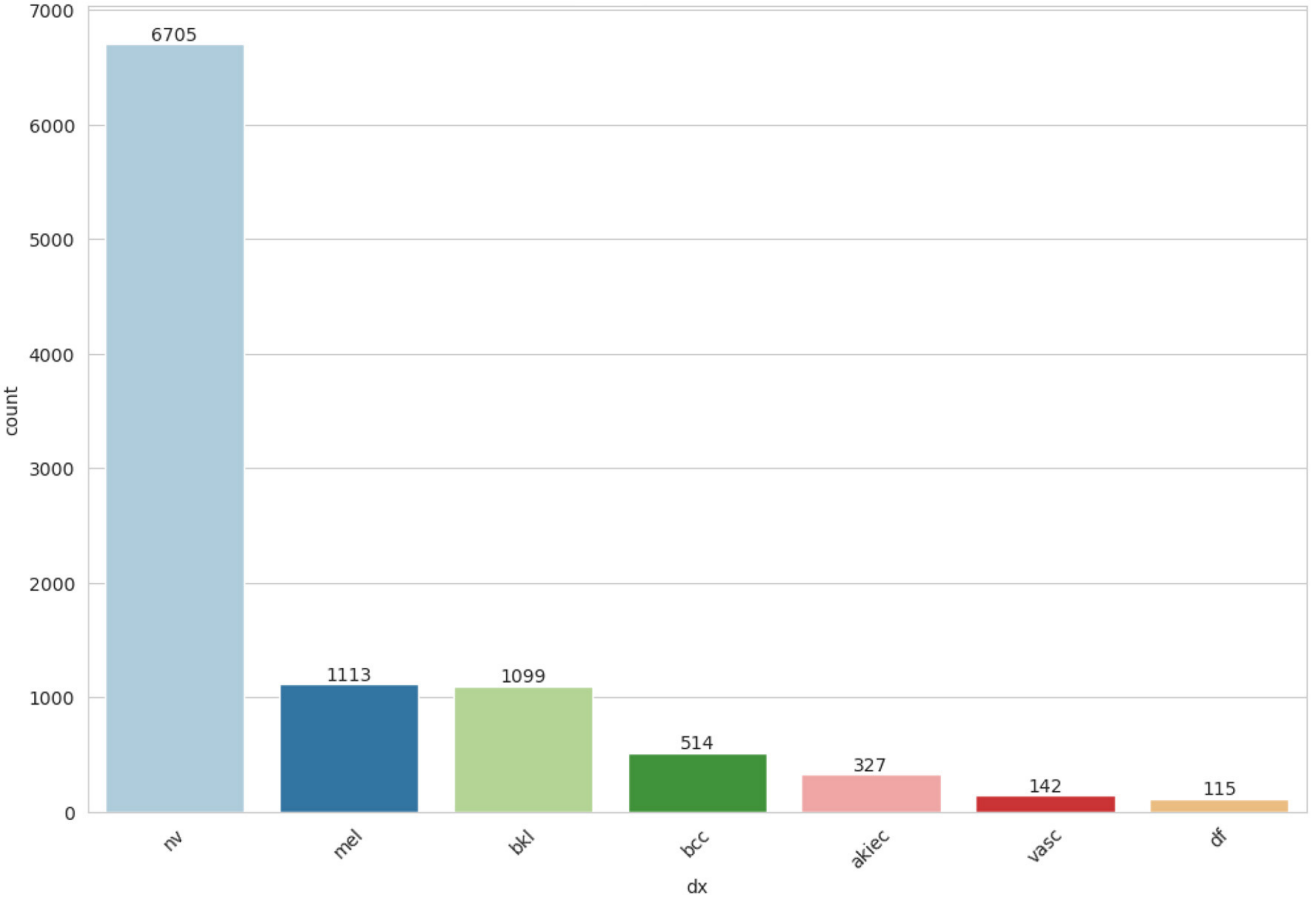
Cell types skin cancer affected patients.

From *meta*_*data* feature matrix, identify the skin cancer cell types that affect the number of patients represented in Figure 4.

**Figure 5:**
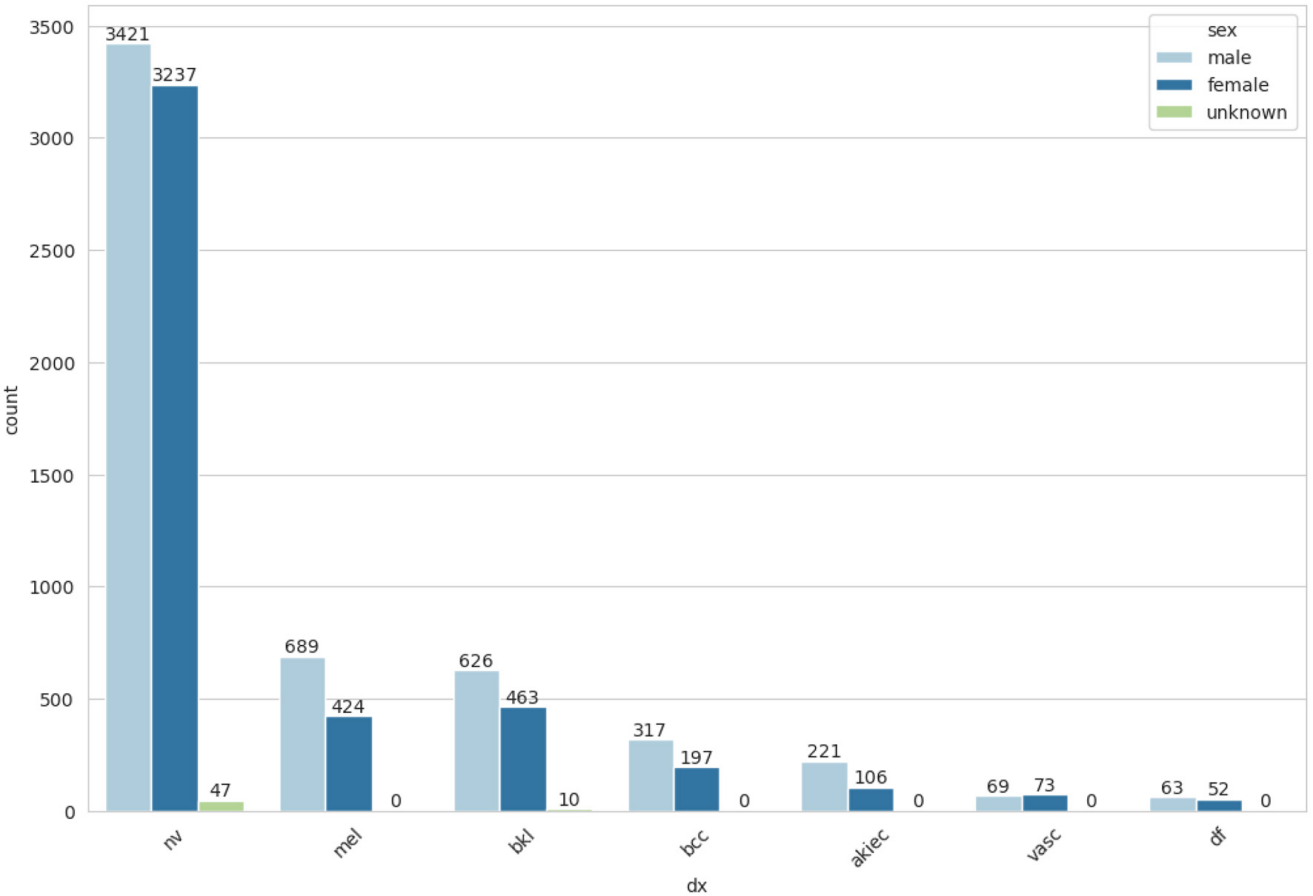
Cell types frequencies.

The frequency of cell types by gender-wise distribution is represented in Figure 5. After performing the EDA, the next step is to balance the dataset by using random oversampling.

#### 3.2.3 Random over sampling

Random oversampling is an ML technique for unbalanced dataset handling. A dataset that has significantly more samples in one class than another is said to be imbalanced. This imbalance could lead to an ML model that is biased against the majority class. To balance the dataset, random oversampling entails randomly multiplying instances from the minority class. This approach replicates the minority class of samples that naturally exist without adding any new information. The feature set in the dataset is *Y*, and x is the label set with classes *C*_1_ (minority class) and *C*_2_ (majority class). In the minority class *C*_1_, the total number of instances is *m*_*C*_1__, and the total number of instances *m*_*C*_2__ is in the majority class *C*_2_. Using random oversampling, the objective is to duplicate instances from *C*_1_ to bring *m*_*C*_1__ equal to *m*_*C*_2__ (24–26) as can be seen in Equations 1–3.

**Initial level imbalance:**


(1) $$\begin{array}{l} m_{C_{1}}<<m_{C_{2}} \end{array}$$

**Target following oversampling:**


(2) $$\begin{array}{l} m_{c_{1}}^{\prime }<<m_{c_{2}} \end{array}$$

Where $n_{c_{1}}^{\prime }$ is the new samples set for the minority class after oversampling.

**Duplication and random selection:** The total samples to duplicate is $m_{C_{1}}^{\prime }-m_{C_{2}}$. If *N*_*C*_1__ indicates the samples set in class *C*_1_. Where $N_{C_{1}}^{\prime }$ is the new samples set for the minority class after random oversampling.


(3) $$\begin{array}{l} N_{C_{1}}^{\prime }=N_{C_{1}}\cup \text{randomsample}\left(N_{C_{1}},m_{C_{2}}-m_{C_{1}}\right) \end{array}$$

#### 3.2.4 Data splitting

To evaluate the interpretation of DL models, the dataset is split into test, validation, and training sets. This prevents overfitting and aids in determining the models' degree of generalizability. The HAM10000 dataset was first split into 80% training and 20% testing sets for this inquiry. For the training and testing split, the following formula was applied mathematically:

*Training set size*


(4) $$\begin{array}{l} X_{train}=round\left(X\times trainratio\right) \end{array}$$

*Test set size*


(5) $$\begin{array}{l} X_{test}=X-X_{train} \end{array}$$

Equations 4, 5 display the training and testing sizes. X is the total number of occurrences in the dataset. Both train_ratio and test_ratio are the ratio of instances to the training set and the proportion of instances to the test set, respectively. These equations give the dimensions of the training and test sets based on the designated ratios. Changing the train_ratio will change the training set's size directly, but changing the test set's size after delegating it to the training set will change its size implicitly.

### 3.3 Models selection

The process of selecting a model includes selecting the appropriate neural network design, optimization strategies, and hyperparameters. Configure hyperparameters for batch size, learning rate, dropout rate, number of layers, activation parameters, and so forth. Hyperparameter tweaking can have a big impact on the model's performance. Each model architecture is trained on the training set using a distinct set of hyperparameters and the pertinent evaluation criteria. This study employed a number of DL models, including the original CNN, the CNN without batch normalization, the CNN with minimal filters, and the strides CNN model, to categorize and forecast skin cancer cases.

### 3.4 Convolutional Neural Network

Convolutional Neural Networks (CNN) use convolutional layers to filter inputs and extract useful information. Convolutional filters are connected to the input via CNN's convolutional layers to calculate the output of the neurons connected to specific input regions. It facilitates the extraction of an image's temporal and spatial properties. The three main layers that comprise the CNN model are the convolutional layer, max-pooling layer, and fully connected layer. The convolutional layer is composed of three crucial parameters: pitch, padding, and filter size. Each layer uses a variety of filters to extract detailed features. The convolutional layer applies a filter (or kernel) across the input image to produce feature maps (Equation 6).


(6) $$\text{Output}(k,l,h)=\sum_{i=1}^{a}\sum_{j=1}^{b}\sum_{c=1}^{C}Y_{i,j,c,h}\cdot \text{Input}(k+i-1,l+j-1,c)+b_{h}$$

The output value at location (*k, l*) in the *h*_*it*_ feature map is denoted by the expression *Output*(*k, l, h*). For channels *c* and filter *h*, the weight of the filter at location (*I, j*) is *CY*_*i, j, c, h*_. The input value at location (*k*+*i*−1, *l*+*j*−1, *c*)+*b*_*h*_ for channel *c* is denoted as *Input*(*k*+*i*−1, *l*+*j*−1, *c*)+*b*_*h*_. *b*_*h*_ is the bias term for filter *h*, *C* is the number of input channels, and *aXb* is the filter's size.

Stride claims the filters are moving inside the images. CNN performs worse when the value is more than two. The stride size is either one or two. In cases where the convolutional layer's filter partially obscures the input images, zero padding is necessary to preserve the structural assessment. Every convolutional layer has a distinct purpose; for example, the first layer draws attention to the margins of the lesions, the second layer extracts intricate geometric details, and the third layer draws attention to the shapes and colors of the lesions. The ReLU layer passes positive values in the feature map, whereas negative values are suppressed and converted to zero (27). To reduce the dimensionality of the collected features, utilize the max-pooling layer (Equation 7). The two most widely used techniques for the max-pooling layer are the max and average.


(7) $$\begin{array}{l} \text{Output}\left(a,b,c\right)=\max_{\left(i,j\right)\in \text{window}}\text{Input}\left(a+i,b+j,c\right) \end{array}$$

The max operation is applied over a predetermined window (e.g., 2 × 2) in the input data, where *Output*(*a, b, c*) is the output value at position (*a, b*) in channel *c*, and *Input*(*a*+*i, b*+*j, c*) refers to the input values within the pooling window. The image is classified into many classes using the fully connected layer (Equation 8) (28, 29).


(8) $$\begin{array}{l} {\text{Output}}_{h}=\overset{M}{\underset{j=1}{\sum }}W_{j,h}\cdot {\text{Input}}_{j}+b_{h} \end{array}$$

Where the *h*−*th*neuron's output in the fully connected layer is denoted by *Output*_*h*_, the weight that connects the *j*−*th* input neuron to the *h*−*th* output neuron is denoted by *W*_*j, h*_, and the input value from the *j*−*th* neuron in the preceding layer is denoted by *Input*_*j*_. There are a total of *N* input neurons. The bias term for the *h*−*th* neuron is *b*_*h*_. Feature map normalization is accomplished using the batch normalization layer as given in Equation 9.


(9) $$\hat{y}=\frac{y-\mu }{\sqrt{\sigma^{2}+\epsilon }}\text{Output}=\gamma \hat{y}+\beta$$

**Figure 6:**
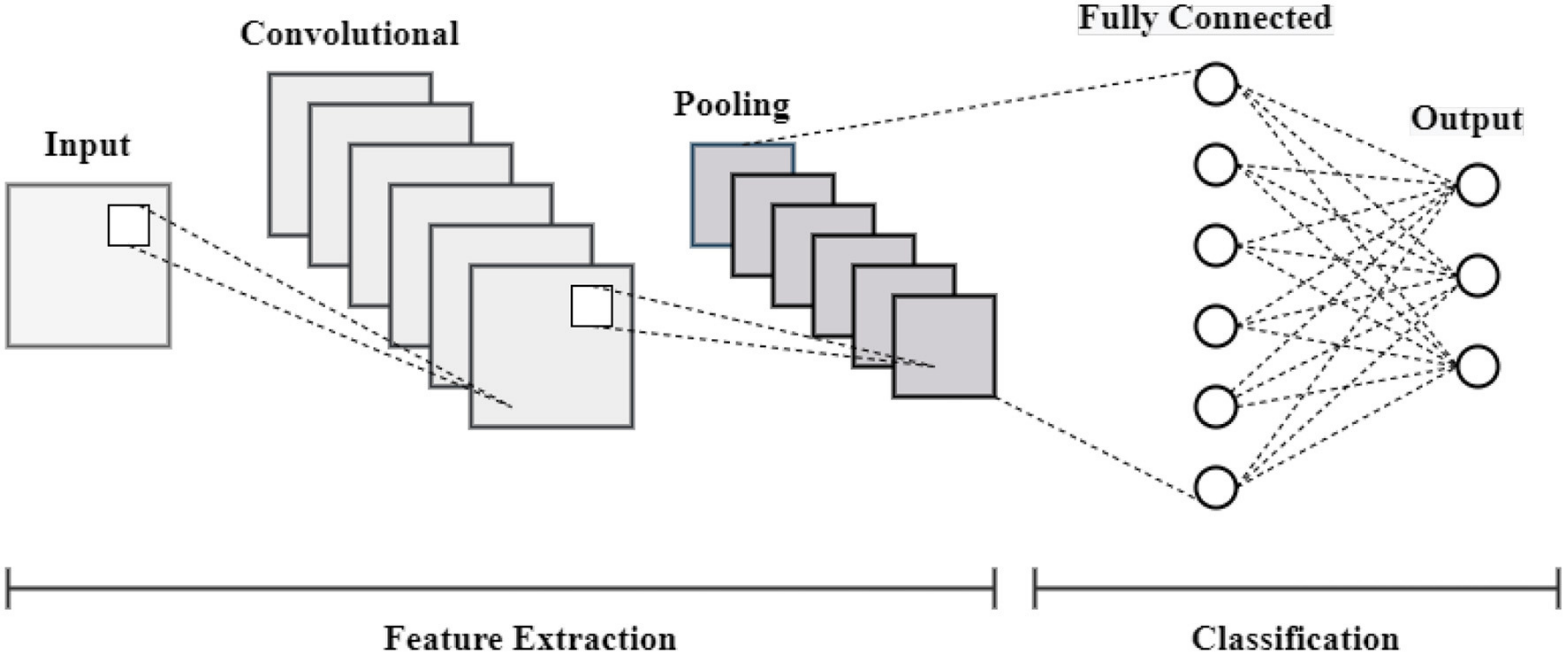
Convolutional neural network architecture.

The input batch mean and variance are denoted by μ and σ^2^, respectively, where *y* is the input value. A tiny constant called ϵ is added for numerical stability. Learnable parameters γ and β shift and scale the normalized value. These tiers expedite network regulation and training. Dropout layers (Equation 10) are used in certain problems and are quite helpful in resolving over-fitting problems in networks (30).


(10) $${\text{Output}}_{i}=\{\begin{array}{ll} \frac{{\text{Input}}_{i}}{1-p} & \text{with probability }(1-p)\\ 0 & \text{with probability }p \end{array}$$

where the input value for the *i*−*th* unit is denoted by *Input*_*i*_. The output value for the *i*−*th* unit following dropout is denoted by *Output*_*i*_. The dropout rate, or the likelihood of setting a unit to zero during training, is denoted by *p*. The architecture of the CNN model is represented in Figure 6.

**Figure 7:**
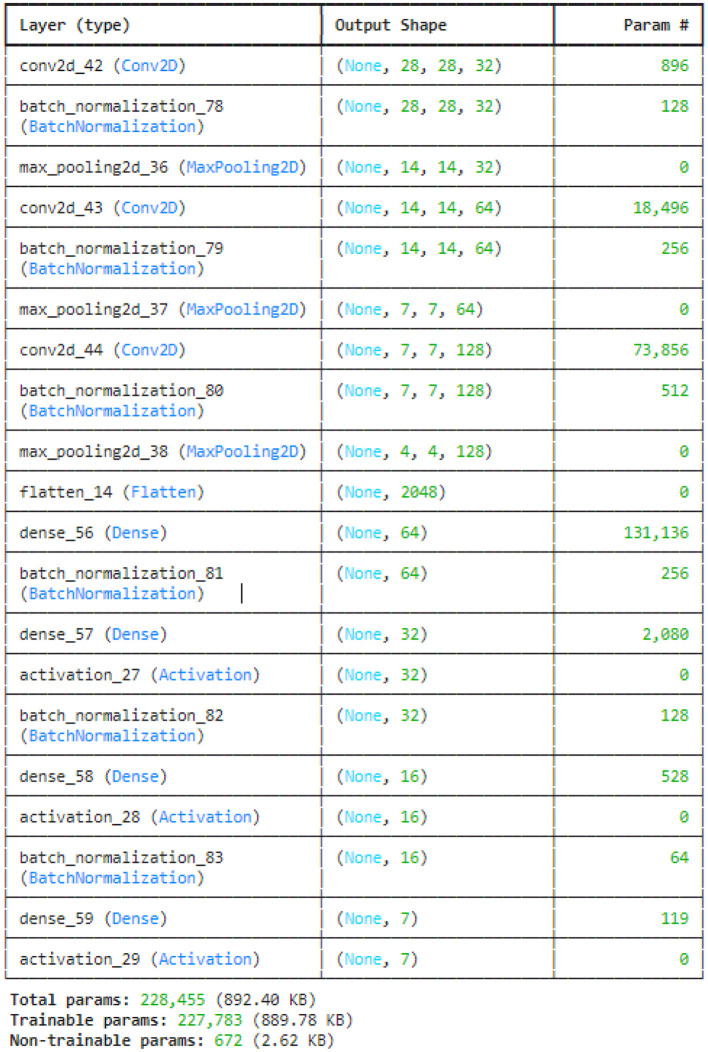
CNN model summary.

This study utilized the four variants of CNN models: original CNN, no batch normalization, few filters and strided CNN model. The original CNN model summary is provided in Figure 7. Sequential CNN architecture includes information on the kind of layer, output shape, and a number of parameters. Multiple layers, including Conv2D, BatchNormalization, MaxPooling2D, Flatten, Dense, and Activation, are included in the model. The MaxPooling2D layers decrease spatial dimensions, while the Conv2D layers gradually increase the depth of the data. After a flattened layer, Dense layers are applied to reduce the data to a lower-dimensional space for final classification. BatchNormalization layers are used to normalize outputs. With 227,783 of the model's total 228,455 parameters being trainable, the complexity and learning potential of the model are demonstrated. Runtime statistics are maintained by non-trainable parameters, which are frequently present in BatchNormalization layers.

**Figure 8:**
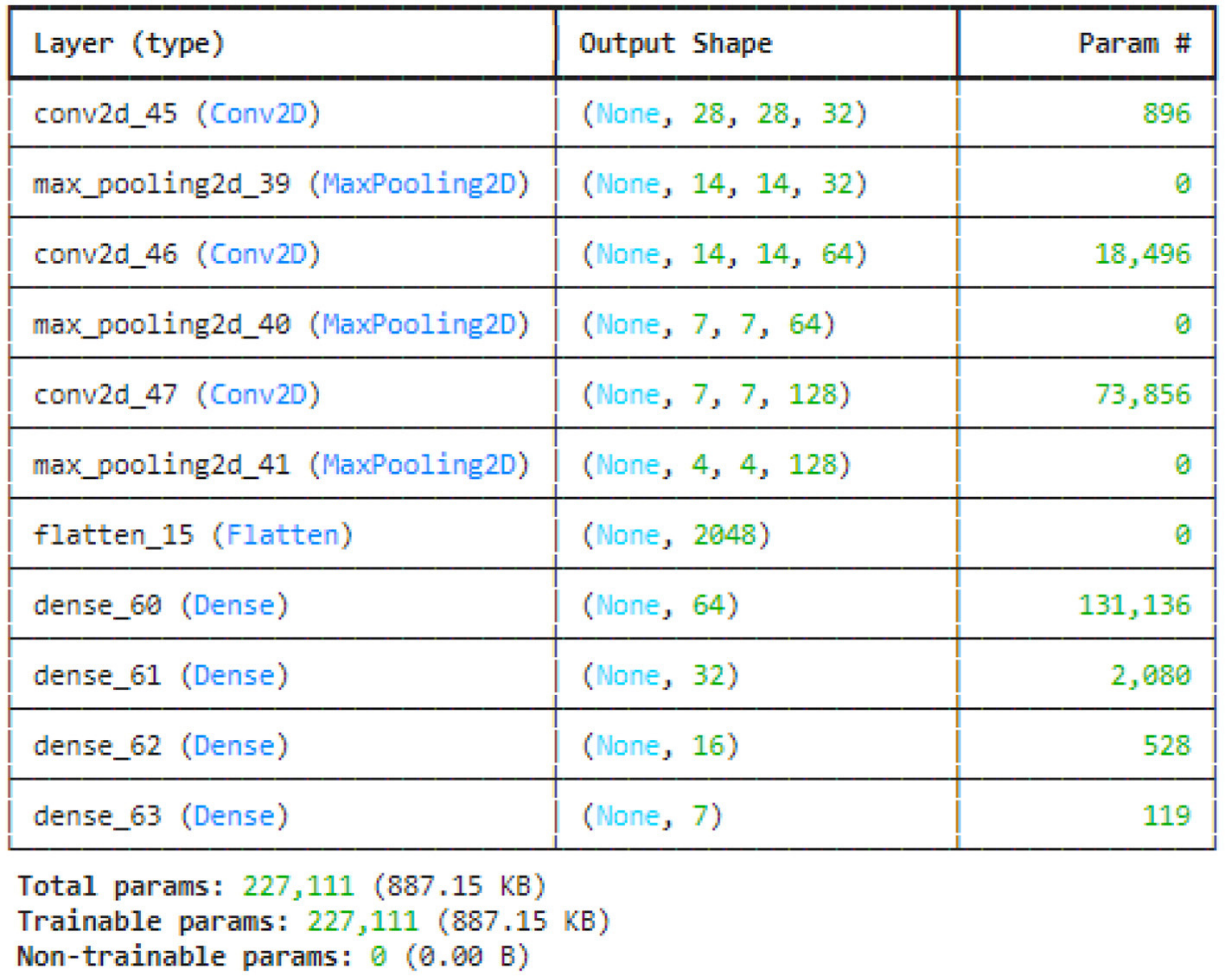
NO batch CNN model summary.

Figure 8 illustrated the no batch normalization CNN model. This CNN model uses multiple convolutional layers to extract features, then pooling layers to reduce dimensionality, flattening the output before processing it through fully connected dense layers for classification. It does not have batch normalization layers, which are usually used to speed up and stabilize training. All of the model's parameters are trainable, meaning that it can learn everything from the data without any non-trainable elements.

**Figure 9:**
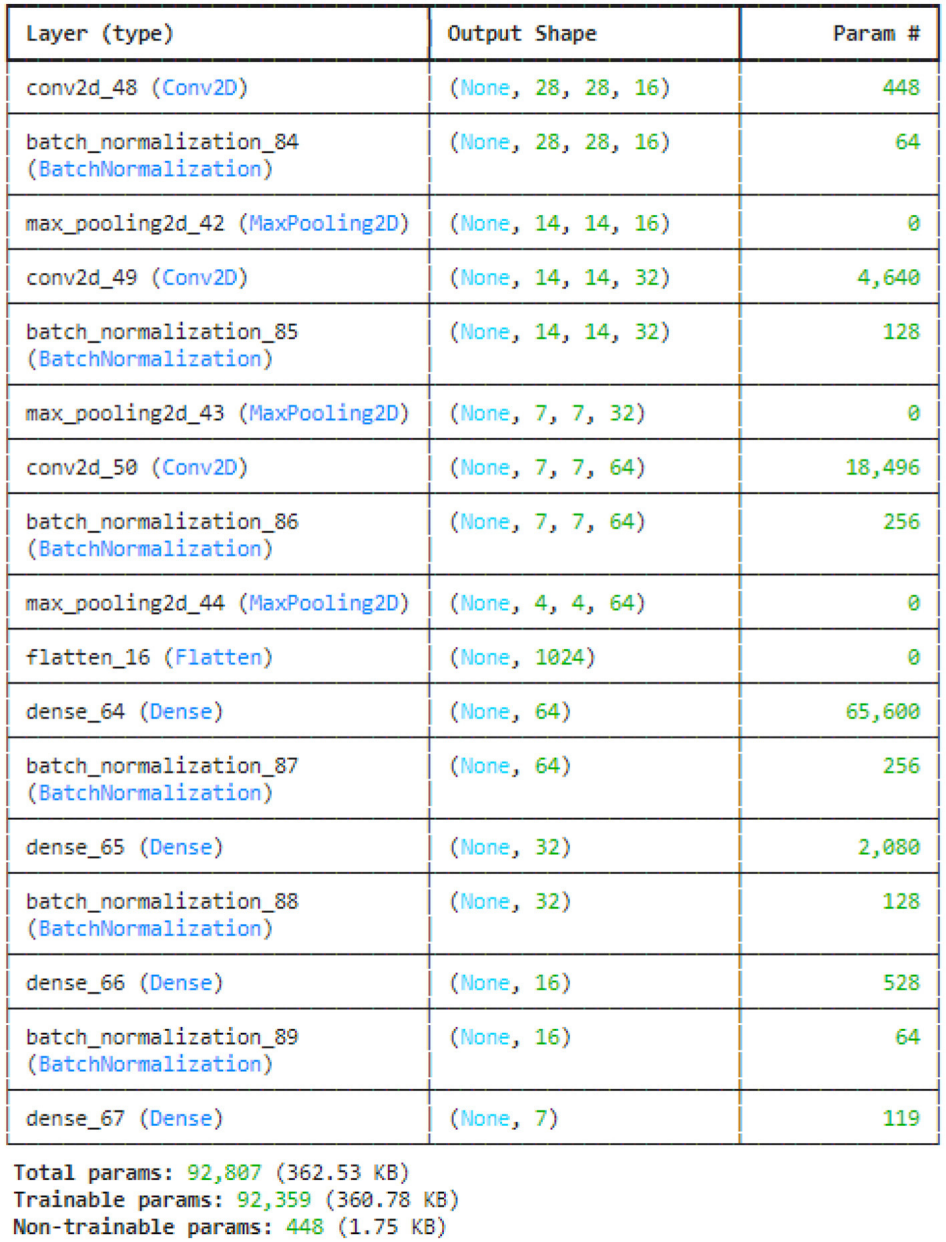
Few filters CNN model summary.

The few filters CNN model is represented in Figure 9. However, with the balance of less complexity and capacity, the model can be trained more quickly with fewer filters. Batch normalization layers follow all convolutional and dense layers to stabilize the training process. Completing multi-class classification tasks is appropriate for this model because its final fully connected layer corresponds to seven output classes. Merely a minor portion linked to the batch normalization layers is untrainable, whereas the rest of the parameters are trainable.

**Figure 10:**
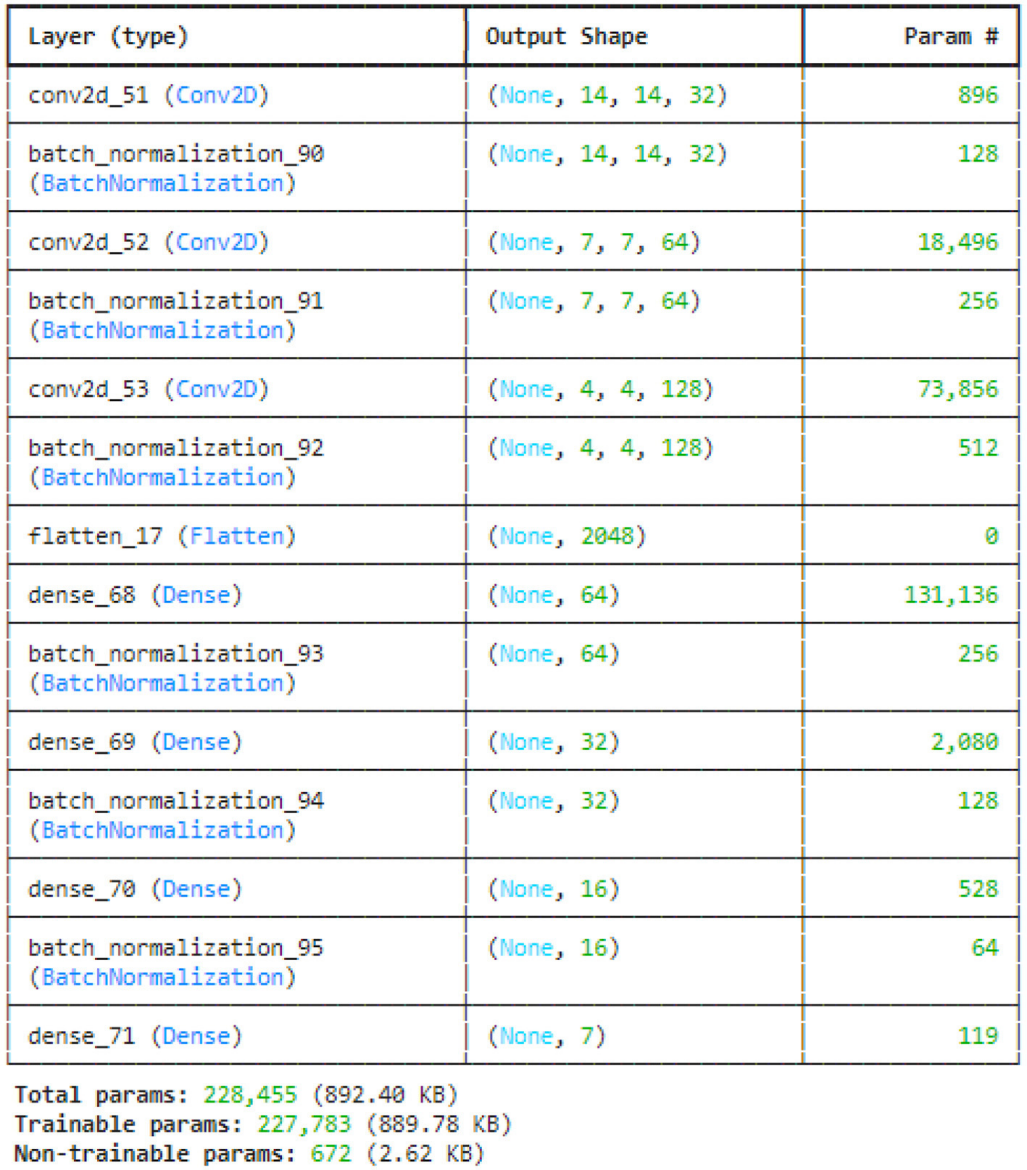
CNN strided model summary.

Figure 10 illustrated the sequential strided CNN model. To minimize the spatial dimensions and go away with the necessity for separate pooling layers, the model employs the use of strided convolutional layers. This makes feature extraction more effective. Each convolutional and dense layer is followed by batch normalization layers that accelerate and stabilize the training procedure. A fully linked layer with seven units is the result appropriate for a seven-class classification problem. Because there are so few non-trainable parameters associated with the batch normalization layers, the model is able to derive a lot from the data.

#### 3.4.1 Feature extraction from CNN model

The goal is to take an existing CNN model and truncate it by deleting a certain number of layers from the end. CNN-based models extract significant features without human inspection. The DL advantage allows for the most effective feature extraction from the training dataset using convolutional filters. After extracting the features, the CNN model is integrated with ML model SVM, RF, and LR using grid search for best parameters.

*Grid Search:* Grid search is an ML methodology that methodically explores every potential value within a predetermined grid to get the optimal set of hyperparameters for a model. The model's performance is greatly impacted by hyperparameters, which are variables that are established prior to training, such as the learning rate or number of layers. A variety of possible values are defined, models are trained for each combination, and their performance is assessed. Cross-validation is frequently used during this procedure to ensure dependability. Although grid search is thorough and simple to use, it can be computationally expensive, particularly when working with large datasets or several hyperparameters. Options such as Bayesian optimization or random search can be more efficient since they concentrate on the most promising regions of the hyperparameter space.

**Figure 11:**
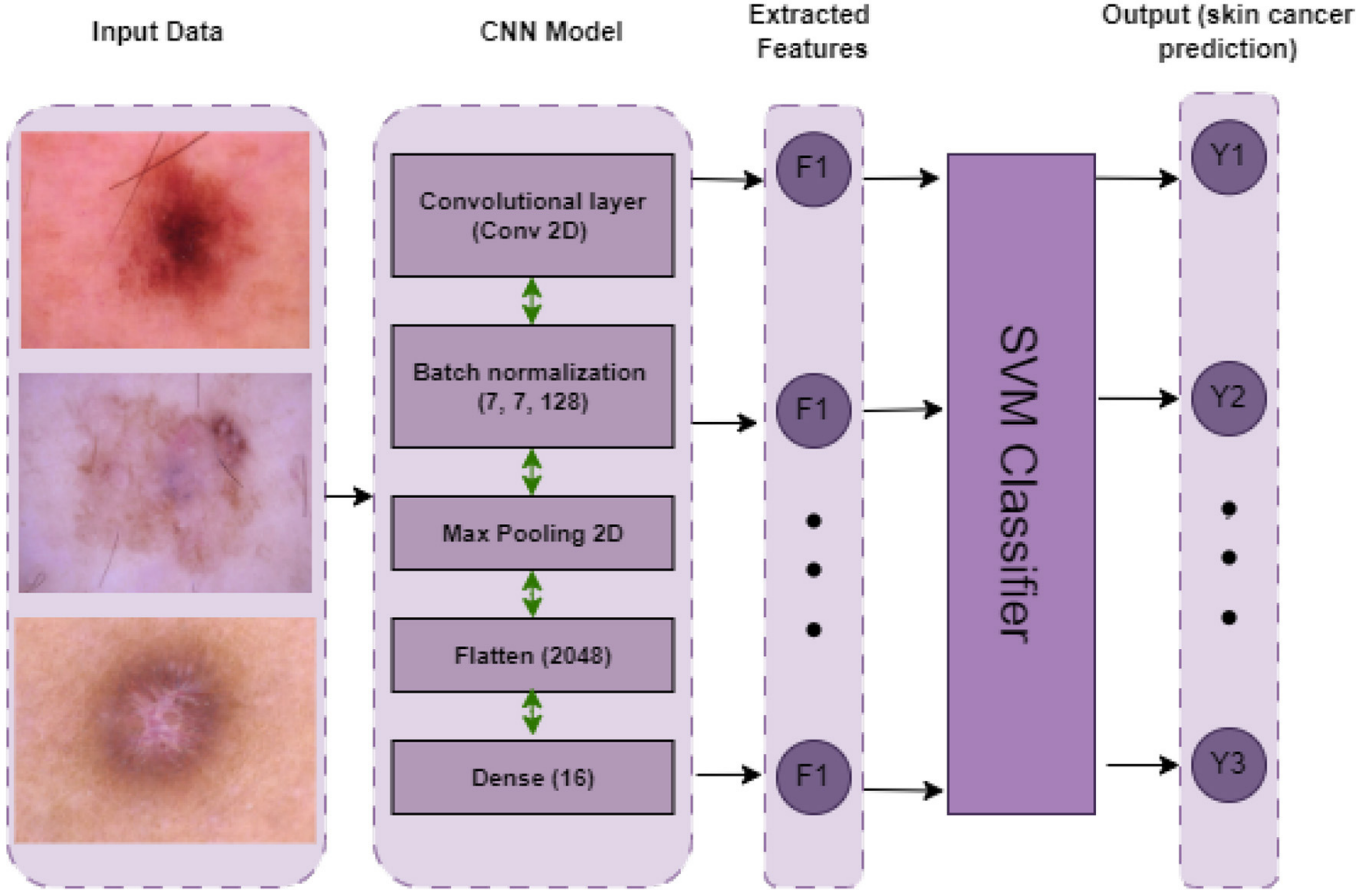
Architecture of CNNSVM model.

*CNNSVM:* Use cross-validation to train and assess an SVM classifier on a dataset. Following cross-validation, the model is tested on a different test set and trained on the full training set. The primary assessment parameter is accuracy. The hyperparameters *C* and γ are set to specified values in the SVM model, which employs the RBF kernel. The architecture of the CNNSVM model is represented in Figure 11.

*CNNRF:* Employs cross-validation to train and assess an RF classifier on a dataset. Following cross-validation, the model is tested on a different test set and trained on the full training set. The RF model is configured with specific hyperparameters that control the number of features considered at each split, the smallest amount of samples required to divide a node, the depth of the trees, and the overall amount of trees in the forest. The evaluation demonstrates the model's performance on both unseen test data and during cross-validation.

**Figure 12:**
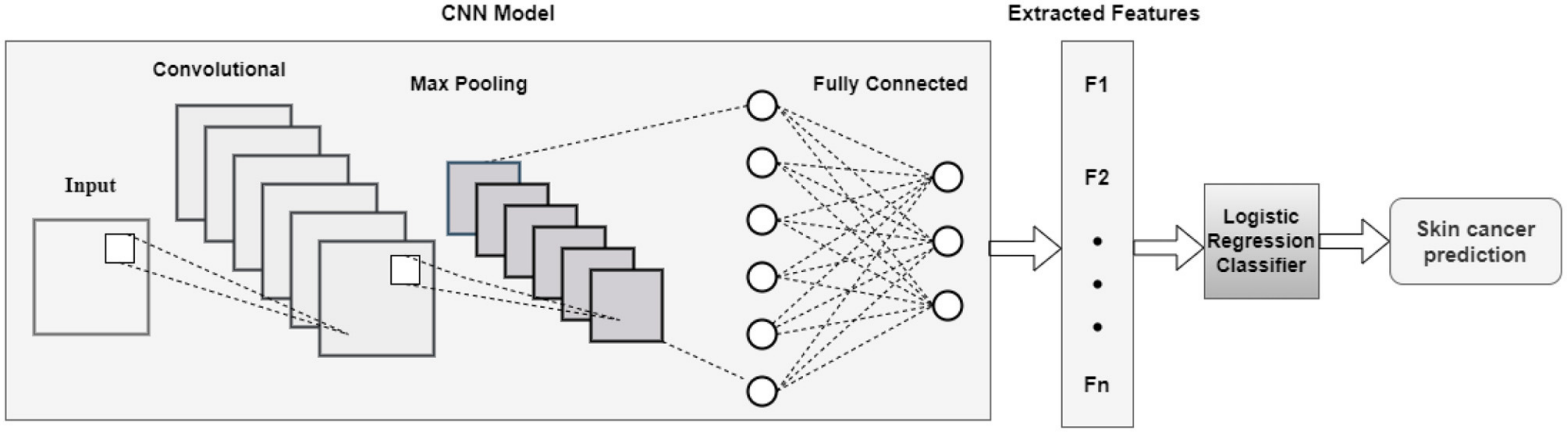
Architecture of CNNLR model.

*CNNLR:* Use cross-validation on a dataset to train and assess an LR classifier. Certain hyperparameters are set up in the model to regulate regularization and complexity. After assessing the model's performance through cross-validation, the complete training set is used for training. The architecture of the CNNLR model is represented in Figure 12.

Algorithm 1 begins with the loading of the HAM10000 dataset, which is made up of tagged images of skin lesions. It then explains how to forecast skin cancer with a deep-learning approach. Preparing the data involves cleaning it, analyzing it exploratorily, and balancing the dataset by randomly oversampling. Next, training and testing sets of data are created from the material. An extractive feature map into a high-dimensional space is made using a CNN model for the classification of skin lesions. Grid search for the best hyperparameters is used to build hybrid models that combine CNN with SVM, RF, and LR. Cross-entropy is used to calculate the loss as the model is trained over a number of epochs and batches. At each batch, predictions are made. The training model, performance metrics, and test predictions are returned as part of the final output, which is assessed using evaluation metrics.

**Algorithm 1:**
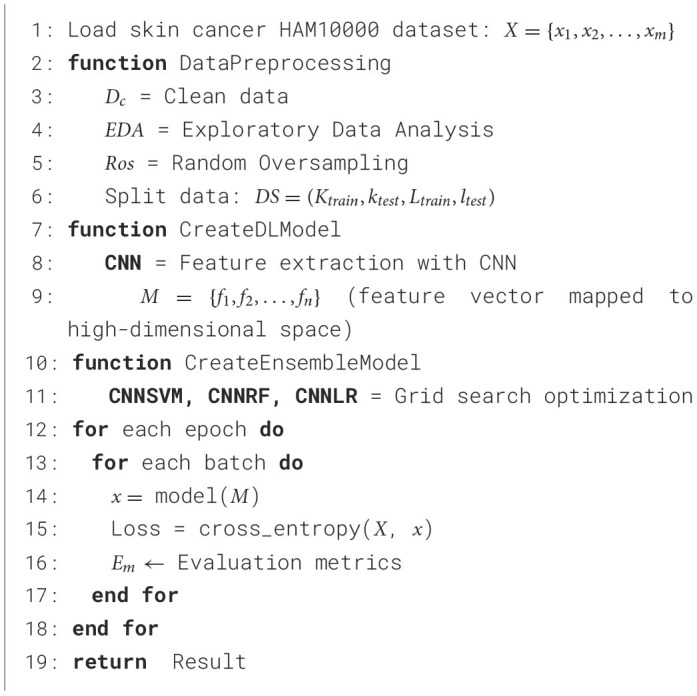
Pseudo code for skin cancer prediction.

## 4 Experimental results and analysis

The evaluation and assessment of experimental findings and interpretation from experiments or data gathered as part of a study. This study assesses the efficiency of the framework using a variety of evaluation criteria, each of which offers valuable insights into the model's workings.

The investigations in this study were conducted using a predetermined set of instruments and technology. The development environment used was Jupyter Notebook, which is well-known for its intuitive interface and capacity to facilitate interactive programming, particularly with Python. Version 3.8.8 of Python was used, which provides a stable and effective environment for Windows applications to operate properly. The computational architecture was based on a laptop with an HP Core i5 CPU, which, although being a mid-range system, provided sufficient power for the experimental activities. When combined with significant memory resources, the processor's capabilities allowed the model to function well even in situations where more processing was needed. Several metrics were used to assess the framework's performance, providing valuable data about model accuracy, processing speed, and general usefulness. Through consistent dependability and efficiency throughout the study, this configuration guaranteed that the hardware and tools met the needs for carrying out challenging deep learning tasks.

### 4.1 Evaluation metrics

This research evaluates the framework's effectiveness based on a wide range of evaluation criteria, all of which provide insightful insights into how the model functions. Accuracy, the first parameter, is usually used as the benchmark for assessing performance. Based on the overall amount of the sample, it is calculated as the portion of accurately identified samples. Equation 11 highlights the parameter's simplicity yet its significant influence, simplifying the process. The degree to which a model or system predicts the positive class is indicated by its accuracy. It is a representation of the model's accuracy and the level of trust placed in its capacity to generate exact forecasts.


(11) $$\begin{array}{l} Acc=\frac{TP+TN}{TP+TN+FP+FN} \end{array}$$

***Precision:***

The accuracy with which a model or system predicts the positive class is defined as its precision. It symbolizes both the model's accuracy and the level of trust in its capacity to generate accurate forecasts. The metric fundamental equation is made easier to understand by showing this value proportionately in Equation 12.


(12) $$\begin{array}{l} Precision=\frac{TP}{TP+FP} \end{array}$$

***Recall:***

The ratio of each positive occurrence to the percentage of exact positive forecasts is the focal point of the evaluation metric known as recall, also referred to as sensitivity. Equation 13's computation demonstrates the special benefit of this balanced viewpoint for estimation.


(13) $$\begin{array}{l} Recall=\frac{TP}{TN+FN} \end{array}$$

***F1-Score:***

The appropriately identified F1 score functions as an equilibrium of memory and precision because it can effectively communicate the essence of a balanced performance. Combining these two metrics yields the F1-score, a popular estimate of model performance that is especially useful for evaluation. This basic estimating procedure is well described by Equation 14, which looks complicated but provides much information.


(14) $$\begin{array}{l} F1-score=2\times \frac{Precision+Recall}{Precision+Recall} \end{array}$$

The Receiver Operating Characteristic-Area Under the Curve (ROC-AUC) is a useful graphical tool for evaluating the effectiveness of classification algorithms. It presents the trade-off, across different threshold settings, between the genuine positive rate (sensitivity) and the false positive rate (1 - specificity). The loss function measures the difference between the expected and actual values in deep learning, sometimes called the cost function. Because it directs the optimization process, it is essential to the training of neural networks. Reducing this loss function is the objective of training.

Table 2 demonstrates the original CNN model results for skin cancer detection. For Class 0, the model performs flawlessly, displaying a 1.00 F1-Score, Precision, and Recall. In Class 2, the model obtains a balanced F1-Score of 0.96 with a Precision of 0.93, suggesting that 93% of the predicted positives are right, and a Recall of 0.99, meaning it correctly detects 99% of real instances. Class 4 shows that the model occasionally misses real positives, as evidenced by the somewhat lower F1-Score of 0.92 despite the Precision staying high at 0.99 and the Recall falling to 0.86. The model performs admirably in Classes 1, 3, 5, and 6. With support values of 1,318, 1,351, and 1,358, respectively, the Precision, Recall, and F1-Scores for Classes 1, 3, and 5 are all perfect at 1.00, demonstrating perfect prediction and identification of instances inside these classes. Despite a minor decline in precision, the model for Class 6 maintains a high recall of 0.99 and a precision of 0.94, producing a strong F1-Score of 0.97. One thousand three hundred and sixty five is the support number for Class 6.

**Table 2:** Original CNN model result.

**Labels**	**Precision**	**Recall**	**F1-Score**	**Support**
0	1.00	1.00	1.00	1,359
1	0.99	1.00	1.00	1,318
2	0.93	0.99	0.96	1,262
3	1.00	1.00	1.00	1,351
4	0.99	0.86	0.92	1,374
5	1.00	1.00	1.00	1,358
6	0.94	0.99	0.97	1,365
**Accuracy**	-	-	0.98	9,387
**Weighted Avg**	0.98	0.98	0.98	9,387

Table 3 indicates variation in performance across classes for the CNN model without batch normalization. With respect to Class 0, the model's precision (Precision 0.94%) is quite high, but its recall (0.63%) is low, meaning that the F1-Score is 0.76%. Class 1's F1-Score of 0.74%, Precision of 0.71%, and Recall of 0.77% indicate a respectable level of balance. Class 2 is a problem for the model; the F1-Score is 0.62%, and the Precision is poor at 0.52%, even with a Recall of 0.77%. With an F1-Score of 0.88% and a high Precision of 0.98%, it does well in Class 3. With a precision of 0.60%, recall of 0.68%, and an F1-Score of 0.64% for Class 4, the model performs moderately. With an F1-Score of 0.92%, the model predicts Class 5 (Precision 1.00%) with high accuracy, but it does neglect some cases. With a Precision and Recall of roughly 0.64% for Class 6, the model's performance is moderate but balanced, yielding an F1-Score of 0.64%. With a Macro Average of 0.77% for Precision, 0.73% for Recall, and 0.74% for F1-Score, the same as the Weighted Average, the overall accuracy is 73%.

**Table 3:** No batch norm CNN model classification result.

**Labels**	**Precision**	**Recall**	**F1-Score**	**Support**
0	0.94	0.63	0.76	1,359
1	0.71	0.77	0.74	1,318
2	0.52	0.77	0.62	1,262
3	0.98	0.81	0.88	1,351
4	0.60	0.68	0.64	1,374
5	1.00	0.86	0.92	1,358
6	0.64	0.63	0.64	1,365
**Accuracy**	-	-	0.73	9,387
**Weighted Avg**	0.77	0.73	0.74	9,387

The performance of the CNN model with fewer filters varies depending on the class provided in Table 4. It yielded an F1-Score of 0.87% for Class 0, where it attains flawless precision but misses certain occurrences. Class 1 has an F1-Score of 0.84%, indicating great precision. Class 2 has trouble with accuracy but makes up for it with a 0.88% recall rate and an F1-Score of 0.79%. With a flawless recall and an F1-Score of 0.95%, the model performs remarkably well for Class 3. Class 4's F1-Score of 0.77% indicates that it has a reasonable balance. Class 5 obtains an F1-Score of 0.96%, indicating excellent overall performance and flawless precision. Class 6 has an F1-Score of 0.81% because of its strong recall and lower precision. With both macro and weighted averages for precision, recall, and F1-score at 0.86%, the total model accuracy is 86%.

**Table 4:** Fewer filters CNN model classification results.

**Class**	**Precision**	**Recall**	**F1-Score**	**Support**
0	1.00	0.78	0.87	1,359
1	0.99	0.72	0.84	1,318
2	0.72	0.88	0.79	1,262
3	0.91	1.00	0.95	1,351
4	0.82	0.72	0.77	1,374
5	1.00	0.93	0.96	1,358
6	0.70	0.97	0.81	1,365
**Accuracy**	-	-	0.86	9,387
**Weighted Avg**	0.88	0.86	0.86	9,387

Table 5 demonstrates the strided CNN Model classification result. All classes exhibit near-perfect precision, recall, and F1-scores, indicating the model's highly accurate performance. Class 0 and Class 3 exhibit perfect recall, precision, and F1-scores of 1.00%. Class 1 and Class 5 also exhibit excellent performance, with F1-scores approaching 1.00% and near-perfect precision of 0.98% and 1.00%, respectively, due to great recall. In addition, Class 2 and Class 6 exhibit good performance, with F1-scores of 0.97% and 0.96% resulting from somewhat lower precision (0.96% and 0.92%) but high recall. Class 4's precision of 0.99% indicates good performance, but its recall of 0.86% yields a somewhat lower F1-score of 0.92%. The model's overall accuracy is 0.98, while its precision, recall, and F1-score macro and weighted averages are all 0.98, demonstrating steady and reliable performance across the dataset.

**Table 5:** Strided conv model classification result.

**Class**	**Precision**	**Recall**	**F1-Score**	**Support**
0	0.99	1.00	1.00	1,359
1	0.98	1.00	0.99	1,318
2	0.96	0.99	0.97	1,262
3	1.00	1.00	1.00	1,351
4	0.99	0.86	0.92	1,374
5	1.00	0.99	1.00	1,358
6	0.92	0.99	0.96	1,365
**Accuracy**	-	-	0.98	9,387
**Weighted Avg**	0.98	0.98	0.98	9,387

**Figure 13:**
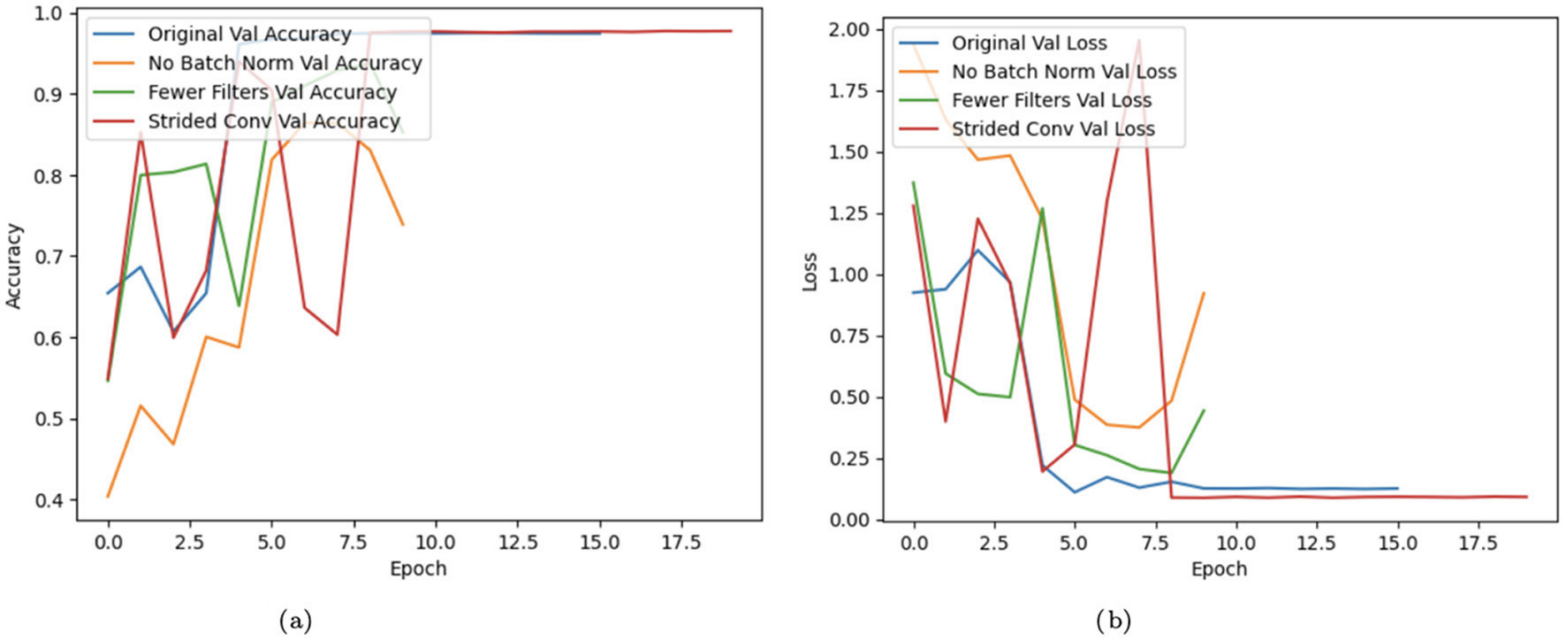
Graphical visualization of CNN model. **(a)** Training graph. **(b)** Loss graph.

A Convolutional Neural Network (CNN) model's training performance is displayed graphically in Figure 13, with an emphasis on validation accuracy and loss over a number of epochs. The X-axis represents the number of epochs, and validation accuracy and loss are displayed in two distinct subplots on the Y-axis. The original CNN model, one without batch CNN, one with fewer filters, and one employing strided convolutions are all contrasted in the graph. Batch normalization exhibits improvements in stability and convergence, whereas fewer filters and strided convolutions, depending on their implementation, may result in poorer accuracy and higher loss. Over time, the accuracy of training generally increases, and the loss is generally reduced.

**Figure 14:**
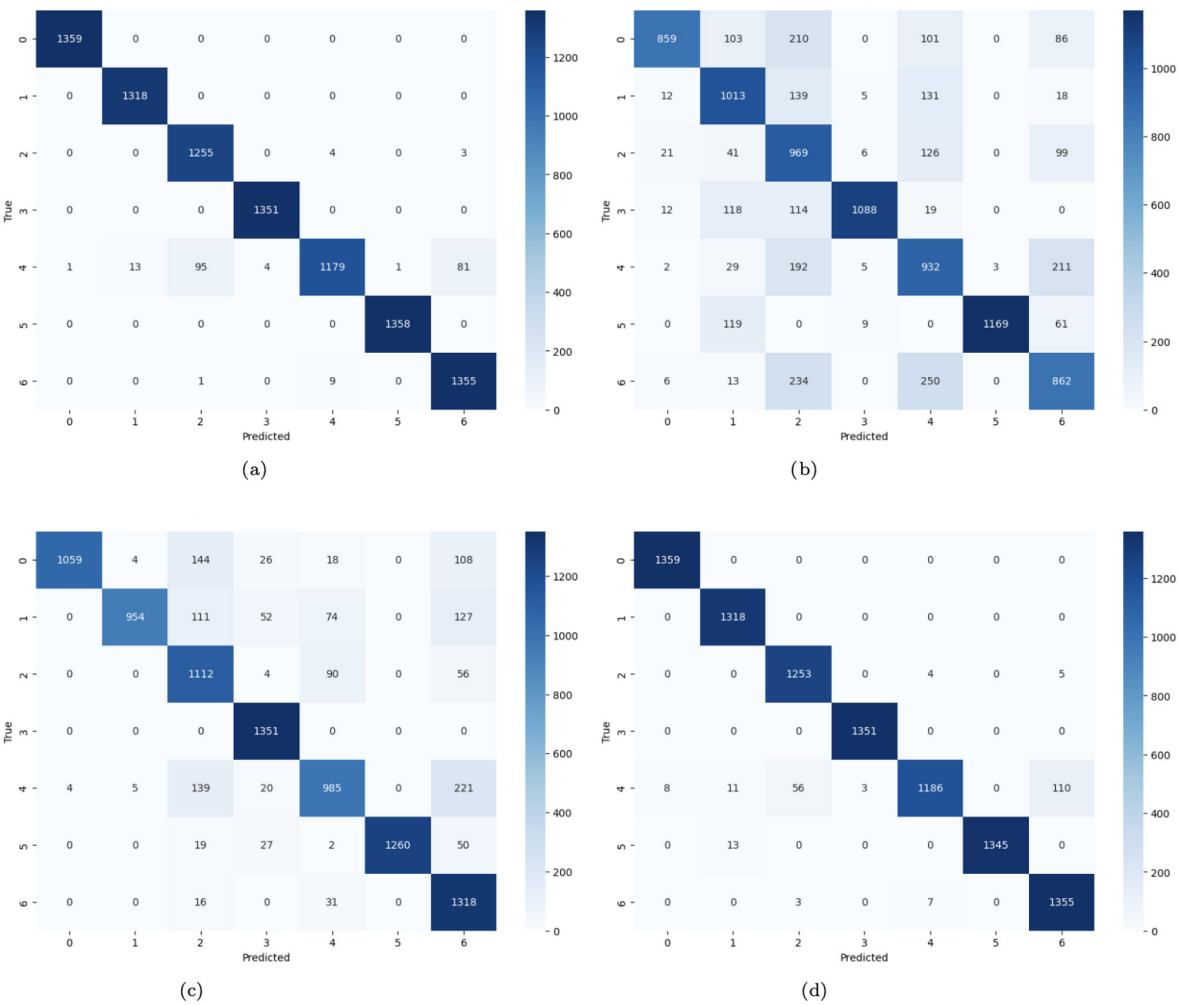
**(a)** Confusion matrix of CNN; **(b)** no batch CNN confusion; **(c)** confusion matrix of fewer filters CNN model; **(d)** strided CNN model confusion matrix for skin cancer detection on HAM10000 dataset.

In Figure 14, the confusion matrices of four distinct CNN models for the prediction of skin cancer are provided. Figure 14a demonstrates that there are many accurate predictions for class 0, 1,359 cases, 1,318 cases for class 1, 1,351 for class 3, and 1,358 skin cancer cases are predicted correctly. For class 2, 1,255 cases are predicted accurately, while 7 cases are misclassified. Similarly, 1,179 cases are diagnosed correctly, and 195 cases are misdiagnosed. For class 6, 1,355 cases are predicted correctly, and 9 cases are misclassified. Figure 14b represents the no-batch CNN model where numerous instances on the diagonal are accurate, and a large fraction of the projections on the off-diagonal are incorrect. Figure 14c represents the few filters CNN model where numerous instances on the diagonal are accurately predicted (i.e., 1059 instances for class 0, 954 for class 1, 1,112 cases for class 2, 1,351 instances for class 3, 985 skin cancer cases for class 4, 1,260 and 1,318 cases for class 5 and 6 respectively). A large fraction of the instances on the off-diagonal are misdiagnosed. Figure 14d demonstrates the strided CNN model where numerous instances on the diagonal are accurately predicted, and few cases on the off-diagonal are misclassified.

**Figure 15:**
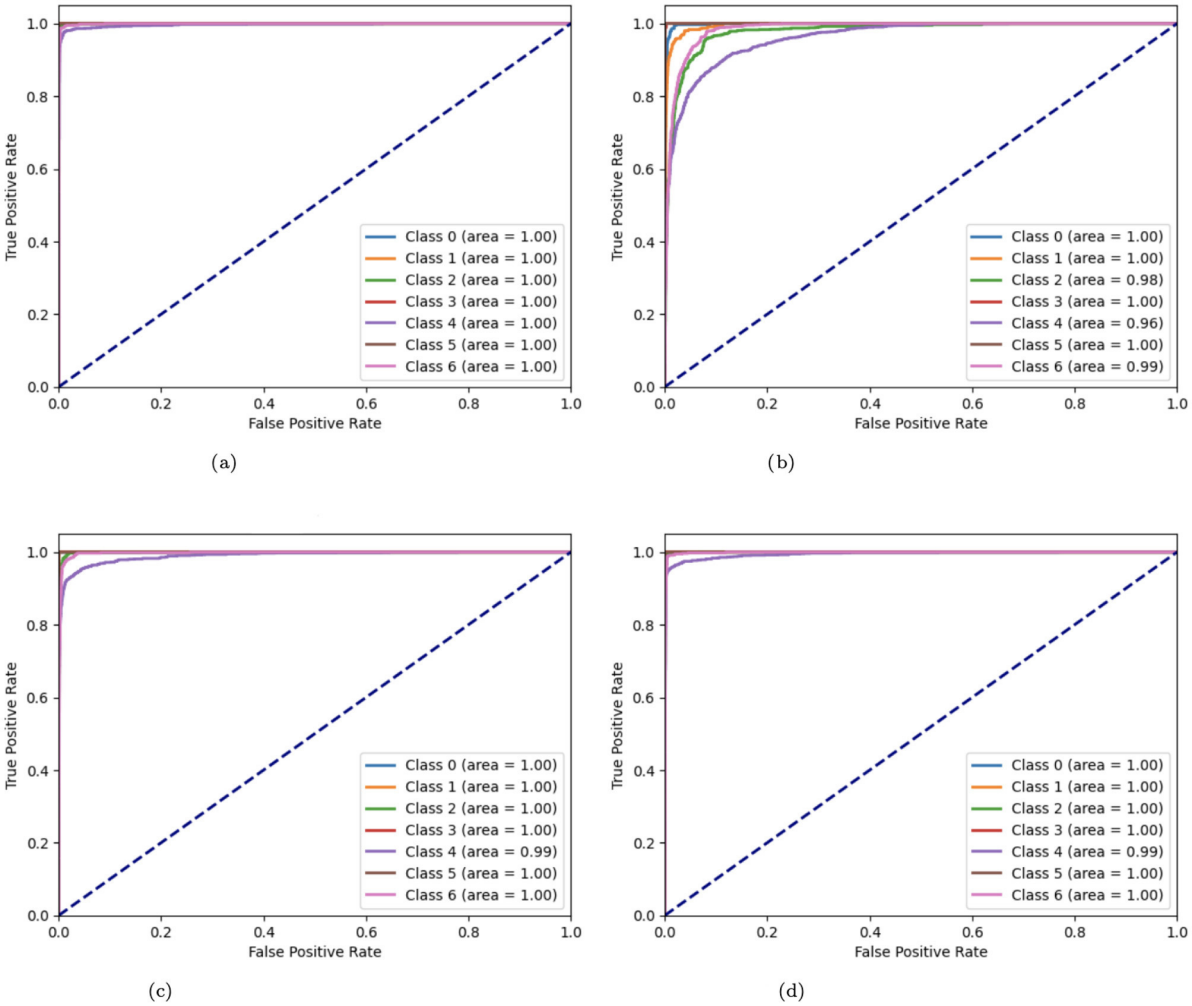
**(a)** ROC curve of CNN; **(b)** no batch CNN ROC curve; **(c)** ROC curve of fewer filters CNN model; **(d)** strided CNN model ROC curve for skin cancer detection on HAM10000 dataset.

The Receiver Operating Characteristic (ROC) curves of variant CNN models are illustrated in Figure 15. ROC curves are produced by plotting the true positive rate (TPR) against the false positive rate (FPR). These curves are used to display the effectiveness of CNN algorithms. A curve that approaches the upper-left corner indicates better model performance. A number of CNN model modifications, including the original, without batch CNN model, with fewer filters, and with strided convolutions, are compared by means of their ROC curves. The original CNN usually performs well, but the aberrations of other variations (such as curves further from the top-left corner) suggest potential issues such as lower complexity learning or loss of spatial information.

Table 6 compares the results of three integrated models (CNNSVM, CNNRF, and CNNLR) utilizing metrics for classification using a dataset on skin cancer. The CNNSVM model demonstrates nearly perfect classification performance for various kinds of skin cancer, such as Actinic keratoses, Basal cell carcinoma, Dermatofibroma, and Pyogenic granulomas. Despite having far lower F1 scores (0.97%) for melanoma and benign keratosis, the model still exhibits good recall and precision. Melanocytic nevi have an F1-Score of 0.94 and a Recall that is marginally lower at 0.91%. The model's overall accuracy is 98%, with both the Macro and Weighted averages of Precision, Recall, and F1-Score at 0.98%. The CNNRF model yields impressive classification results, with good Precision, Recall, and F1-Score (1.00%) for most kinds of skin cancer, including Actinic keratoses, Basal cell carcinoma, Dermatofibroma, and Pyogenic granulomas. Both benign keratosis and melanoma perform well, with F1-Scores of 0.98%, because of their good precision (0.96%) and outstanding recall (1.00%). Melanocytic nevi has a 0.95% F1-Score because of its high Precision and somewhat poor Recall (0.91%). The weighted and macro averages of the model are both 0.99%, suggesting consistently strong performance across all classes, and its overall accuracy is 99%. The CNNLR model demonstrates exceptional classification performance for actinic keratoses, basal cell carcinoma, dermatofibroma, and pyrogenic granulomas, with perfect Precision, Recall, and F1-Scores (1.00%). Melanoma and benign keratosis both perform well, with precision values of 0.96 and 0.95 and F1-scores of 0.97%, respectively. Melanocytic nevi's F1-Score is 0.94% because of its higher Precision of 0.98 and lower Recall of 0.91%. Overall, the model has a 99% accuracy rate; its macro and weighted averages are 0.99%, and it regularly performs well in all forms of skin cancer.

**Table 6:** Integrated models result.

**Validation**	**Labels**	**Precision**	**Recall**	**F1-score**	**Support**
CNNSVM	Actinic keratoses	1.00	1.00	1.00	1,359
	Basal cellcarcinoma	0.99	1.00	1.00	1,318
	Benign keratosis	0.96	0.99	0.97	1,262
	Dermatofibroma	1.00	1.00	1.00	1,351
	Melanocytic nevi	0.98	0.91	0.94	1,374
	Pyogenic granulomas	1.00	1.00	1.00	1,358
	Melanoma	0.95	0.99	0.97	1,365
	Accuracy	-	-	0.98	9,387
	Weighted avg	0.98	0.98	0.98	9,387
CNNRF	Actinic keratoses	1.00	1.00	1.00	1,359
	Basal cellcarcinoma	0.99	1.00	1.00	1,318
	Benign keratosis	0.96	1.00	0.98	1,262
	Dermatofibroma	1.00	1.00	1.00	1,351
	Melanocytic nevi	1.00	0.91	0.95	1,374
	Pyogenic granulomas	1.00	1.00	1.00	1,358
	Melanoma	0.96	1.00	0.98	1365
	Accuracy	-	-	0.99	9,387
	Weighted avg	0.99	0.99	0.99	9,387
CNNLR	Actinic keratoses	1.00	1.00	1.00	1,359
	Basal cellcarcinoma	0.99	1.00	1.00	1,318
	Benign keratosis	0.96	0.99	0.97	1,262
	Dermatofibroma	1.00	1.00	1.00	1,351
	Melanocytic nevi	0.98	0.91	0.94	1,374
	Pyogenic granulomas	1.00	1.00	1.00	1,358
	Melanoma	0.95	0.99	0.97	1,365
	Accuracy	-	-	0.99	9,387
	Weighted avg	0.99	0.99	0.99	9,387

Table 7 represents the comparison of the proposed methodology with existing studies. The study by Andleeb et al. (17) revealed an accuracy of 75.5% in the comparison of different models for skin cancer diagnosis, which is quite low compared to other models. With an accuracy of 92.5%, the research by Harish et al. (13) achieved a noteworthy improvement. The accuracy was further improved to 93.8% by Naeem et al. (6), suggesting that better feature integration or the adoption of a more complex model were factors in the higher performance. In contrast, Chaturvedi et al.'s model (19) had an accuracy of 90.7%. According to Shapna et al. (20), the model performed admirably, achieving 91.3% accuracy. With an astounding accuracy of 99%, the proposed model in this analysis performs noticeably better than compared to current methods.

**Table 7:** Comparison with existing techniques.

**References**	**Accuracy**
Andleeb et al. (17)	75.5%
Harish et al. (13)	92.5%
Naeem et al. (6)	93.8%
Chaturvedi et al. (19)	90.7%
Shapna Akter et al. (20)	91.3%
Proposed model (CNNLR)	99 %

### 4.2 Findings and discussion

Deep learning models are used to analyze and interpret medical images in order to look for symptoms of diseases like skin cancer. Abnormalities in demographic images are identified using this method. The experiment shows that the proposed model has a satisfactory performance on the HAM10000 image dataset. The effectiveness of the proposed model is evaluated using statistical analytic techniques. Statistical analysis is used to evaluate the DL model outputs for effectiveness, generalizability, and application. “Model complexity” describes the degree of complexity in a model's structure and ability to find patterns and correlations in the image data while discussing DL. To determine its level of complexity, a DL model's architecture uses a variety of factors, including weights and biases. A model's complexity grows with the amount of parameters provided, and this is mostly expressed in the number of layers, neurons per layer, and connections between them. The ability of the model to recognize complex patterns and correlations in the data is increased with each further layer or neuron by adding new weights and biases to be learned. The model becomes more expressive as the number of parameters increases, which enables it to represent functions that are progressively more complex. However, there is a cost to this enhanced expressiveness: in order to train and produce predictions, the model needs more memory and processing capacity, which increases its computational cost. This parameter diversity improves the model's capacity to identify intricate patterns. Still, it also increases the possibility of overfitting, particularly when the model begins to learn from the training set rather than make generalizations from it. When a model performs well on training data but is unable to predict fresh, unseen data correctly, this is known as overfitting. The tendency of large models with numerous parameters to fit the training data too closely is a typical problem in DL. Regularization techniques, including dropout, weight decay (L2 regularization), and batch normalization, are used to reduce overfitting in DL models. By normalizing each layer's output, batch normalization increases training stability and speed. To prevent excessive weights, weight decay adds a penalty to the loss function, which helps prevent the model from being too complex. During training, dropout randomly eliminates neurons to help the model learn more resilient, universal properties. When combined, these methods aid in balancing model complexity and enhance performance on untested data. Deep Learning models (CNN, no batch normalization, few filters and strided CNN) can enhance the model's performance. To solve the problem of classifying demographic images, this work employs DL models. The experiment results show that the proposed DL model performs more accurately and efficiently than conventional techniques. The test findings show that the proposed model performs better than other techniques for predicting skin cancer.

## 5 Conclusion

This article presented four variants of the CNN model (i.e., original CNN, no batch normalization CNN, few filters CNN, and strided CNN) for the classification and prediction of skin cancer in lesion images with the aim of helping physicians in their diagnosis. The study also presented the hybrid model CNN-SVM, CNN-RF, and CNN-LR, using a grid search for the best parameters for the classification. The findings demonstrate that the proposed approach (CNNLR), which uses the patient's metadata (i.e., the lesion's anatomical site, age, and gender) as the model's input data, enhances the performance of skin lesion classification by at least 5%. The study concludes that DL models, especially different CNN architectures and hybrid models, hold significant promise in enhancing the accuracy of skin cancer diagnosis. The proposed approaches, which surpass traditional diagnostic methods, achieve high accuracy rates by using lesion images combined with patient metadata. The study acknowledges the ongoing need for a clinically sound and widely applicable method of skin cancer screening despite these promising findings. Future work in skin lesion categorization can focus on integrating metadata with image-based deep-learning algorithms. CNN and recurrent neural networks (such as LSTM or BiLSTM) used in hybrid models might be used to extract correlations between temporal metadata and visual attributes. More sophisticated hybrid methods like CNN-XGBoost, CNN-SVM, and CNN-Random Forest could enhance resilience. Critical areas of an image might be given priority by attention processes or Transformer-based models, which would also dynamically weigh the information. Model adaptation to new datasets can be improved by meta-learning techniques like Model-Agnostic Meta-Learning (MAML). Finally, simultaneous processing of images and metadata using multimodal deep learning architectures may enhance classification performance and clinical usefulness.

## Data Availability

The original contributions presented in the study are included in the article/supplementary material, further inquiries can be directed to the corresponding authors.
